# Combined television viewing and computer use and mortality from all-causes and diseases of the circulatory system among adults in the United States

**DOI:** 10.1186/1471-2458-12-70

**Published:** 2012-01-23

**Authors:** Earl S Ford

**Affiliations:** 1 Division of Adult and Community Health, National Center for Chronic Disease Prevention and Health Promotion, Centers for Disease Control and Prevention, Atlanta, GA, USA; 2 Centers for Disease Control and Prevention, 4770 Buford Highway, MS K67, Atlanta, GA 30341, USA

**Keywords:** Mortality, Sedentary lifestyle, Television

## Abstract

**Background:**

Watching television and using a computer are increasingly common sedentary behaviors. Whether or not prolonged screen time increases the risk for mortality remains uncertain.

**Methods:**

Mortality for 7,350 adults aged ≥ 20 years who participated in the National Health and Nutrition Examination Survey during 1999-2002 and were followed through 2006 was examined. Participants were asked a single question about the amount of time they spent watching television or videos or using a computer during the past 30 days.

**Results:**

During a median follow-up of 5.8 years, 542 participants died. At baseline, 12.7% of participants reported watching television or using a computer less than 1 h per day, 16.4% did so for 1 h, 27.8% for 2 h, 18.7% for 3 h, 10.9% for 4 h, and 13.5% for 5 or more h. After extensive adjustment, the hazard ratio for all-cause mortality for the top category of exposure was 1.30 (95% confidence interval: 0.82, 2.05). No significant trend across categories of exposure was noted. The amount of screen time was also not significantly related to mortality from diseases of the circulatory system.

**Conclusions:**

In the present study, screen time did not significantly predict mortality from all-causes and diseases of the circulatory system.

## Background

During the first quarter of 2010, the typical American watched over 35 h of television per week as well as over 2 h of time shifted television and used the internet for almost 4 h [1]. According to the 2010 American Time Use Survey, people aged ≥ 15 years watched television an average of 2.7 h per day making it the most common leisure activity [2]. Of some concern is that the amount of television viewing has been drifting steadily upwards [1].

Excessive amounts of watching television have been linked in cross-sectional as well as prospective studies to obesity, insulin resistance, metabolic syndrome, diabetes, and other conditions [3-11]. Because of the epidemics of obesity and diabetes and the possible contribution of excessive television viewing to these conditions, curtailing the quantity of television viewing among children has become a Healthy People 2020 objective [12].

Several studies have examined the links between television watching and mortality from all-causes and cardiovascular disease [13-16]. A meta-analysis of this set of studies found that prolonged screen time was modestly associated with an increased risk of all-cause mortality (adjusted hazard ratio [aHR] per 2 h of television viewing per day = 1.13, 95% confidence interval [CI] = 1.07, 1.18) and fatal or nonfatal cardiovascular disease (aHR per 2 h of television viewing per day = 1.15, 95% CI = 1.06, 1.23). Only one of these studies was conducted in the United States and included a selective sample of men [14]. Because watching television and using a computer are such common sedentary behaviors, a thorough understanding about the possible links between the amount of time that people spent watching television or using computers is critical to developing sound recommendations about these sedentary behaviors. Consequently, the objective of this study was to examine the relationship between the time spent watching television or videos and using computers outside of work and mortality in a population-based sample of adults in the United States.

## Methods

This study was based on data from the public files for the 2006 follow-up of participants of the 1999-2000 and 2001-2002 cycles of the National Health and Nutrition Examination Survey (NHANES). A multistage, stratified sampling design was used to generate a sample of participants who were representative of the non institutionalized civilian US population. The response rates for the interviewed and examined samples of the entire survey were 82% (9965/12160) and 76% (9282/12160), respectively, for 1999-2000 and 84% (11039/13156) and 80% (10477/13156), respectively, for 2001-2002. After an interview at home, participants were invited to complete additional questionnaires, undergo a set of tests, and provide blood and other biological specimens in the mobile examination center. Methodological details about the NHANES and the linked mortality files have been published [17,18]. The National Center for Health Statistics Research Ethics Review Board granted approval for the conduct of the study, and participants were asked to sign an informed consent form.

The mortality status of participants aged ≥ 20 years through 2006 was determined by using the National Death Index [18]. Several studies have shown that the National Death Index identifies over 90% of deaths [19-21]. Participants who were not deemed to have died as of December 31, 2006 were considered to be alive. The International Classification of Diseases, 10th Revision (ICD-10) codes I00-I99 were used to identify deaths from diseases of the circulatory system.

Participants were asked "Over the past 30 days, on a typical day how much time altogether did you spend on a typical day sitting and watching TV or videos or using a computer outside of work? Would you say...". Response options were none, less than 1 h, 1 h, 2 h, 3 h, 4 h, or 5 h or more. The time spent watching TV or videos or using a computer will also be referred to as screen time.

Study covariates included age, gender, race or ethnicity (white, African American, Mexican American, and other), educational attainment (< high school, high school graduate or equivalent, > high school), smoking status (current, former, never), leisure-time physical activity (continuous), Healthy Eating Index score (continuous), alcohol use (continuous), self-reported health status, health insurance coverage, histories of cardiovascular disease and cancer, body mass index, systolic blood pressure, and concentrations of high-density lipoprotein cholesterol, non-high-density lipoprotein cholesterol, and HbA1c. Participants who had smoked 100 cigarettes during their lifetime and reported smoking at the time of the interview were classified as current smokers. Participants who had smoked 100 cigarettes during their lifetime and reported not smoking at the time of the interview were classified as former smokers. Participants who had never smoked 100 cigarettes during their lifetime were classified as never smokers. Participants were asked about partaking in moderate and vigorous physical activities in leisure-time and, for those who did, the time spent being physically active was calculated from their responses to the frequency and duration of the reported moderate and vigorous activities with the time spent being vigorously active being weighted by a factor of 2. The Healthy Eating Index is a score that ranges from 0 to 100 and has 10 subcomponents: grains, fruits, vegetables, dairy, meats, fats, saturated fat, cholesterol, sodium, and variety [22]. The index was determined from dietary information collected by a single 24-h recall administered in person to participants attending the medical examination. The intake of alcohol was obtained from information provided during a single 24-h dietary recall.

Self-reported health status was determined from the question "Would you say your health in general is excellent, very good, good, fair, or poor?". Health insurance coverage (yes/no) was derived from the question "Are you covered by health insurance or some other kind of health care plan?". Participants who reported ever being told by a doctor or other health professional that they had congestive heart failure, coronary heart disease, angina pectoris, heart attack, or stroke were considered as having a history of cardiovascular disease. Participants who reported ever being told by a doctor or other health professional that they had diabetes were considered to have diabetes. Participants who reported ever being told by a doctor or other health professional that they had cancer were considered to have cancer.

Body mass index (kg/m^2^) was calculated from measured weight and height. Up to four attempts were made to measure blood pressure. The average of the last two measurements of blood pressure for participants who had three measurements, the last measurement for participants with only two measurements, and the only measurement for participants who had one measurement were used. Serum total cholesterol and high-density lipoprotein cholesterol were measured enzymatically on a Hitachi 704 Analyzer (Roche Diagnostics, Indianapolis, IN) at Johns Hopkins University. Non-high-density lipoprotein cholesterol was calculated by subtracting the concentration of high-density lipoprotein cholesterol from that of total cholesterol. Concentrations of HbA1c were measured on Primus Automated HPLC systems, models CLC330 and CLC385 (Primus Corp., Kansas City, MO) at the University of Missouri-Columbia.

The analyses included participants who were aged ≥ 20 years and nonpregnant women. Chi-square tests and t-tests for independent samples were used to examine differences in percentages and means, respectively. The Cochran-Mantel-Haenzel test was used to test for differences in the distribution of categorical variables after stratification by age groups. Age-adjusted mortality rates per 1,000 person-years of follow-up were calculated. Age-adjustment was performed using the direct method with the projected year 2000 US population. Proportional hazards analysis was used to estimate hazard ratios. Using Schoenfeld residuals, the proportional hazards assumption was met. Several proportional hazards models were run that included varying sets of covariates selected from age, gender, race or ethnicity, educational attainment, smoking status, leisure-time physical activity, Healthy Eating Index score, alcohol use, self-reported health status, health insurance coverage, and histories of cardiovascular disease and cancer. The statistical softwares SAS and SUDAAN were used to generate the results.

## Results

Of the 9,471 participants aged ≥ 20 years who had an examination, 10 were ineligible for follow-up because the information needed to link to the National Death Index was lacking. Of the 9,448 participants who provided information about the amount of screen time, 7,350 participants who had complete data for all the study variables were included in the analyses.

**Figure 1:**
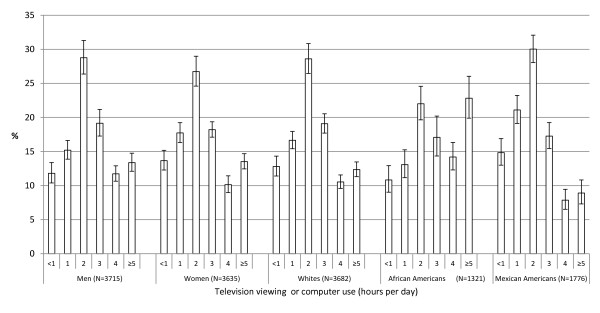
**Age-adjusted distribution (95% confidence interval) of time spent watching television or using a computer among U.S. adults aged ≥ 20 years, by gender and race or ethnicity, National Health and Nutrition Examination Survey 1999-2002**.

After adjustment for age, 12.7% reported no or less than 1 h per day of screen time, 16.4% reported 1 h, 27.8% reported 2 h, 18.7% reported 3 h, 10.9% reported 4 h, and 13.5% reported 5 or more hours. The distributions of the proportions of adults watching television or using a computer differed by gender (*p *= 0.003) and by race or ethnicity (*P *< 0.001) (Figure 1).

Participants were followed on average for 5.8 years during which time 542 deaths including 190 deaths from diseases of the circulatory system were recorded. The unweighted follow-up time of the cohort was 41,502 person-years. Decedents and survivors differed in a number of study variables (Table 1). As screen time increased, significant increases or decreases were noted for all covariates except for reporting a history of cancer and alcohol consumption (Table 2).

**Table 1:** Selected age-adjusted baseline characteristics among adults aged > = 20 years, by mortality status, National Health and Nutrition Examination Survey 1999-2002

	Total (N = 7350)	Deceased (N = 542)	Survivors (N = 6808)	
		
Characteristics	Mean or% (95% CI)	Mean or% (95% CI)	Mean or% (95% CI)	P
Age (years)	45.3 (44.6, 46.0)	66.9 (64.7, 69.0)	44.3 (43.6, 45.0)	< 0.001
Men,%	49.2 (48.1, 50.2)	64.3 (51.5, 75.3)	48.6 (47.4, 49.7)	0.013
Whites,%	73.3 (69.4, 76.8)	63.5 (53.0, 72.9)	73.4 (69.5, 77.0)	0.050
> High school,%	53.1 (50.2, 56.0)	32.2 (23.1, 42.9)	53.7 (50.8, 56.6)	< 0.001
Current smoker,%	24.1 (22.2, 26.1)	33.7 (23.9, 45.1)	23.5 (21.6, 25.6)	0.075
Leisure-time physical activity (min/month)	321.1 (285.1, 357.2)	310.3 (6.6, 613.9)	326.3 (289.6, 362.9)	0.914
Alcohol intake (g)	11.6 (10.1, 13.1)	10.6 (2.9, 18.3)	11.6 (10.1, 13.2)	0.775
Healthy Eating Index score	63.7 (62.9, 64.5)	58.9 (56.8, 60.9)	63.9 (63.0, 64.7)	< 0.001
Body mass index (kg/m^2^)	27.9 (27.6, 28.2)	29.3 (27.5, 31.0)	27.9 (27.6, 28.2)	0.138
Systolic blood pressure (mm Hg)	123.1 (122.3, 123.9)	122.8 (119.0, 126.6)	123.0 (122.2, 123.8)	0.926
High-density lipoprotein cholesterol (mg/dl)	50.9 (50.1, 51.7)	46.5 (43.7, 49.3)	50.9 (50.2, 51.7)	0.002
Non-high-density lipoprotein cholesterol (mg/dl)	152.2 (150.0, 154.3)	167.3 (147.1, 187.4)	151.9 (149.8, 154.1)	0.130
HbA1c (%)	5.4 (5.4, 5.5)	5.9 (5.6, 6.3)	5.4 (5.4, 5.5)	0.006
Excellent or very good health,%	54.7 (52.5, 56.9)	33.3 (24.8, 43.2)	55.8 (53.6, 58.0)	< 0.001
Health insurance coverage,%	82.6 (80.8, 84.4)	80.4 (68.7, 88.4)	82.8 (80.9, 84.5)	0.637
Diagnosed diabetes,%	6.3 (5.7, 7.1)	16.1 (10.8, 23.2)	5.9 (5.1, 6.8)	0.003
History of cardiovascular disease,%	8.3 (7.5, 9.3)	26.3 (17.7, 37.3)	7.4 (6.6, 8.4)	0.001
History of cancer,%	7.9 (7.2, 8.7)	13.3 (8.9, 19.6)	7.5 (6.8, 8.4)	0.038
Screen time (hours per day)				0.025
< 1	12.7 (11.5, 14.0)	5.8 (3.0, 11.0)	12.8 (11.6, 14.1)	
1	16.4 (15.5, 17.3)	10.2 (5.4, 18.3)	16.5 (15.6, 17.3)	
2	27.8 (26.1, 29.5)	34.7 (26.0, 44.6)	28.0 (26.3, 29.7)	
3	18.7 (17.6, 19.9)	20.7 (11.1, 35.4)	18.6 (17.3, 19.9)	
4	10.9 (10.1, 11.9)	7.1 (4.0, 12.3)	11.0 (10.2, 12.0)	
> = 5	13.5 (12.6, 14.4)	21.5 (13.8, 32.0)	13.1 (12.3, 14.1)	

**Table 2:** Selected age-adjusted baseline characteristics among adults aged > = 20 years, by level of screen time, National Health and Nutrition Examination Survey 1999-2002

	Screen time (hours per day)						
	< 1	1	2	3	4	> = 5	
		
Characteristics	Mean or% (95% CI)	Mean or% (95% CI)	Mean or% (95% CI)	Mean or% (95% CI)	Mean or% (95% CI)	Mean or% (95% CI)	P for trend
Age (years)	43.6 (42.6, 44.6)	43.0 (41.7, 44.3)	45.3 (44.3, 46.2)	46.8 (45.4, 48.1)	47.3 (45.8, 48.7)	46.4 (45.4, 47.3)	< 0.001
Men,%	46.1 (42.7, 49.5)	46.4 (43.2, 49.6)	51.1 (48.1, 54.1)	50.7 (47.2, 54.1)	53.0 (49.1, 56.8)	48.6 (45.3, 51.8)	0.012
Whites,%	72.9 (67.3, 78.0)	74.1 (69.8, 78.0)	75.3 (70.7, 79.4)	74.7 (69.9, 79.0)	71.0 (66.1, 75.5)	67.0 (62.3, 71.4)	0.013
> High school,%	58.9 (55.4, 62.4)	58.4 (53.7, 63.1)	54.0 (50.1, 57.9)	52.1 (47.6, 56.5)	47.7 (42.4, 53.2)	43.2 (39.7, 46.7)	< 0.001
Current smoker,%	19.6 (15.6, 24.2)	16.5 (13.4, 20.2)	22.1 (19.0, 25.6)	29.2 (25.6, 33.1)	29.1 (26.4, 31.8)	35.0 (31.6, 38.5)	< 0.001
Leisure-time physical activity (min/month)	443.2 (319.6, 566.8)	336.3 (276.6, 396.0)	337.8 (296.4, 379.1)	323.2 (272.0, 374.4)	257.0 (207.2, 306.9)	198.8 (165.1, 232.5)	0.043
Alcohol intake (g)	10.2 (7.9, 12.4)	12.0 (9.5, 14.5)	12.0 (9.4, 14.6)	10.4 (8.7, 12.0)	13.0 (9.3, 16.7)	12.8 (8.4, 17.3)	0.881
Healthy Eating Index score	65.4 (64.2, 66.6)	65.2 (64.1, 66.4)	64.1 (62.9, 65.3)	62.6 (61.6, 63.5)	63.4 (61.9, 64.8)	60.9 (59.9, 61.9)	< 0.001
Body mass index (kg/m^2^)	26.5 (26.0, 27.0)	27.2 (26.6, 27.9)	27.8 (27.4, 28.2)	28.0 (27.4, 28.7)	29.4 (28.8, 30.1)	28.9 (28.3, 29.5)	< 0.001
Systolic blood pressure (mm Hg)	121.8 (120.5, 123.1)	122.5 (121.3, 123.8)	122.9 (121.8, 124.0)	123.7 (122.5, 124.9)	124.9 (123.0, 126.8)	124.4 (122.9, 125.8)	0.007
High-density lipoprotein cholesterol (mg/dl)	53.0 (52.0, 53.9)	52.3 (50.7, 53.8)	50.9 (49.7, 52.0)	50.4 (49.3, 51.6)	50.0 (48.1, 51.9)	48.9 (47.4, 50.4)	0.001
Non-high-density lipoprotein cholesterol (mg/dl)	147.3 (143.6, 150.9)	149.7 (146.1, 153.3)	153.7 (150.8, 156.6)	153.8 (150.7, 156.9)	153.0 (149.0, 156.9)	155.0 (151.8, 158.2)	0.001
HbA1c (%)	5.4 (5.3, 5.4)	5.4 (5.4, 5.5)	5.4 (5.4, 5.5)	5.5 (5.4, 5.5)	5.5 (5.4, 5.6)	5.6 (5.5, 5.7)	0.039
Excellent or very good health,%	62.7 (59.5, 65.9)	60.7 (54.9, 66.2)	57.1 (54.1, 60.0)	53.5 (50.2, 56.8)	44.2 (39.5, 49.0)	42.8 (38.6, 47.2)	< 0.001
Health insurance coverage,%	83.7 (80.4, 86.5)	84.0 (80.6, 86.9)	84.9 (82.5, 87.1)	82.0 (79.7, 84.1)	81.5 (77.5, 84.8)	75.7 (71.7, 79.3)	0.001
Diagnosed diabetes,%	5.1 (3.6, 7.2)	4.8 (3.3, 6.8)	5.7 (4.7, 6.7)	7.1 (5.4, 9.2)	7.2 (5.8, 8.8)	9.4 (7.4, 11.9)	0.001
History of cardiovascular disease,%	6.5 (4.8, 8.7)	7.4 (5.9, 9.3)	7.7 (6.3, 9.3)	7.9 (6.8, 9.2)	7.4 (6.1, 8.8)	14.8 (12.7, 17.3)	< 0.001
History of cancer,%	7.1 (5.1, 9.6)	7.1 (5.1, 9.8)	7.5 (6.3, 8.9)	8.6 (7.1, 10.5)	7.4 (5.7, 9.6)	9.6 (7.5, 12.3)	0.159

In the model that only adjusted for age, participants reporting 5 or more hours per day of screen time had significantly increased mortality (Table 3). With progressively increasing numbers of covariates, the adjusted hazard ratio decreased steadily. After adjusting for sociodemographic factors and lifestyle behaviors, the attenuated hazard ratio still retained its statistical significance. Additional adjustment for health status, insurance coverage, and prevalent chronic conditions further attenuated the hazard ratio, and the confidence interval of the hazard ratio included the null. The aHR for screen time as a continuous variable adjusted for variables in model 5 in Table 3 was 1.03 per hour (95% CI: 0.97, 1.10) or 1.07 per 2 h (95% CI: 0.93, 1.22) for all-cause mortality and 1.01 per hour (95% CI: 0.88, 1.15) or 1.02 per 2 h (95% CI: 0.78, 1.33) for mortality from diseases of the circulatory system. Adding several possible mediating variables (body mass index, systolic blood pressure, and concentrations of high-density lipoprotein cholesterol, non-high-density lipoprotein cholesterol, and HbA1c) did not further attenuate the hazard ratios. Screen time was not significantly related to mortality from diseases of the circulatory system. To examine possible nonlinearity of screen time, a quadratic term was added to the model but proved to be nonsignificant (*p *= 0.634 for all-cause mortality, *p *= 0.903 for diseases of the circulatory system).

**Table 3:** Sample sizes, rates, and hazard ratios for mortality from all-causes and diseases of the circulatory system among participants aged > = 20 years, National Health and Nutrition Examination Survey 1999-2002-2006

	Screen time (hours per day)						
		
	< 1	1	2	3	4	> = 5	P for trend
**All-causes**							
Unweighted no. deaths/no. at risk	56/910	66/1178	117/2007	106/1360	67/819	130/1076	--
Unweighted person-years	5115	6777	11,504	7713	4611	5782	--
Age-adjusted rate/1,000 PY (95% CI)	8.6 (5.2, 12.1)	8.7 (6.7, 10.8)	8.0 (6.4, 9.6)	10.5 (7.6, 13.4)	8.0 (5.4, 10.7)	13.6 (10.2, 16.9)	--
Model 1 (hazard ratio, 95% CI)	1.00	1.03 (0.67, 1.58)	1.04 (0.74, 1.47)	1.41 (0.79, 2.51)	1.11 (0.69, 1.78)	1.84 (1.19, 2.86)	0.001
Model 2 (hazard ratio, 95% CI)	1.00	1.04 (0.69, 1.57)	1.03 (0.73, 1.46)	1.40 (0.80, 2.44)	1.03 (0.65, 1.63)	1.74 (1.14, 2.64)	0.001
Model 3 (hazard ratio, 95% CI)	1.00	1.07 (0.70, 1.63)	1.05 (0.74, 1.48)	1.35 (0.77, 2.37)	0.97 (0.61, 1.55)	1.55 (1.00, 2.40)	0.024
Model 4 (hazard ratio, 95% CI)	1.00	1.18 (0.76, 1.85)	1.16 (0.81, 1.67)	1.45 (0.83, 2.55)	1.02 (0.64, 1.63)	1.41 (0.90, 2.19)	0.173
Model 5 (hazard ratio, 95% CI)	1.00	1.16 (0.72, 1.85)	1.14 (0.80, 1.61)	1.40 (0.79, 2.47)	1.00 (0.62, 1.62)	1.30 (0.82, 2.05)	0.336
Model 6 (hazard ratio, 95% CI)	1.00	1.10 (0.69, 1.73)	1.09 (0.78, 1.53)	1.38 (0.78, 2.42)	1.02 (0.64, 1.61)	1.33 (0.85, 2.09)	0.181
Model 5: Exclude deaths in year 1	1.00	1.11 (0.69, 1.79)	1.16 (0.81, 1.66)	1.40 (0.77, 2.55)	0.95 (0.58, 1.56)	1.34 (0.81, 2.21)	0.307
**Diseases of the circulatory system**							
Unweighted no. deaths/no. at risk	23/910	23/1178	34/2007	40/1360	24/819	46/1076	--
Unweighted person-years	5115	6777	11,504	7713	4611	5782	--
Age-adjusted rate/1,000 PY (95% CI)	3.2 (2.0, 4.4)	3.5 (1.6, 5.4)	1.9 (1.1, 2.7)	3.5 (2.1, 4.9)	2.2 (1.2, 3.3)	4.1 (2.3, 6.0)	--
Model 1 (hazard ratio, 95% CI)	1.00	1.09 (0.54, 2.19)	0.63 (0.35, 1.14)	1.14 (0.59, 2.21)	0.77 (0.38, 1.58)	1.37 (0.77, 2.44)	0.167
Model 2 (hazard ratio, 95% CI)	1.00	1.13 (0.57, 2.25)	0.66 (0.36, 1.20)	1.19 (0.60, 2.34)	0.77 (0.36, 1.61)	1.33 (0.72, 2.45)	0.263
Model 3 (hazard ratio, 95% CI)	1.00	1.18 (0.56, 2.45)	0.70 (0.37, 1.32)	1.24 (0.60, 2.56)	0.76 (0.36, 1.62)	1.29 (0.64, 2.57)	0.428
Model 4 (hazard ratio, 95% CI)	1.00	1.42 (0.64, 3.17)	0.82 (0.42, 1.59)	1.42 (0.61, 3.30)	0.84 (0.38, 1.83)	1.20 (0.58, 2.49)	0.858
Model 5 (hazard ratio, 95% CI)	1.00	1.35 (0.59, 3.07)	0.83 (0.42, 1.64)	1.50 (0.65, 3.46)	0.88 (0.38, 2.05)	1.14 (0.51, 2.54)	0.909
Model 6 (hazard ratio, 95% CI)	1.00	1.14 (0.56, 2.32)	0.77 (0.43, 1.38)	1.39 (0.69, 2.80)	0.88 (0.39, 1.99)	1.13 (0.57, 2.24)	0.653
Model 5: Exclude deaths in year 1	1.00	1.18 (0.59, 2.38)	0.82 (0.38, 1.73)	1.44 (0.58, 3.58)	0.61 (0.24, 1.50)	1.12 (0.47, 2.65)	0.990

*CI *= confidence interval; *PY *= person-years

Model 1 is adjusted for age

Model 2 is adjusted for age, gender, race or ethnicity, educational attainment

Model 3 is adjusted for variables in model 2 plus smoking status, leisure-time physical activity, Healthy Eating Index score, and alcohol consumption

Model 4 is adjusted for variables in model 3 plus health status and health insurance coverage

Model 5 is adjusted for variables in model 4 plus histories of diabetes, cardiovascular disease, and cancer

Model 6 is adjusted for variables in model 5 plus body mass index, systolic blood pressure, and concentrations of high-density lipoprotein cholesterol, non-high-density lipoprotein cholesterol, and HbA1c

Using model 4 as shown in Table 3 and screen time as a continuous variable, no effect modification by age (< 65 years versus > = 65 years) (*p *= 0.469 for mortality from diseases of the circulatory system), gender (p interaction = 0.809 for all-cause mortality and *p *= 0.281 for mortality from diseases of the circulatory system) or by race or ethnicity for the three major groups (p interaction = 0.721 for all-cause mortality and *p *= 0.568 for mortality from diseases of the circulatory system) was noted. However, the hazards ratios for all-cause mortality differed significantly for participants aged < 65 years (aHR: 1.11, 95% CI: 1.00, 1.23) and > = 65 years (aHR: 1.01, 95% CI: 0.94, 1.08) (p interaction = 0.036). A model that excluded participants who died during the first year (491 total deaths and 169 from diseases of the circulatory system among 7,342 participants) failed to show evidence of a significant association between screen time and all-cause mortality or diseases of the circulatory system.

Hazard ratios were also calculated for a reduced set of categories that are generally consistent with some previous studies (Table 4). The results did not show significant associations between screen time and mortality from all-causes or diseases of the circulatory system.

**Table 4:** Sample sizes, rates, and hazard ratios for mortality from all-causes and diseases of the circulatory system among participants aged > = 20 years, National Health and Nutrition Examination Survey 1999-2002-2006

	Screen time (hours per day)			
	
	< 2	2- < 4	> = 4	P for trend
**All-causes**				
Unweighted no. deaths/no. at risk	122/2088	223/3367	197/1895	--
Unweighted person-years	11,892	19,218	10,393	--
Age-adjusted rate/1,000 PY (95% CI)	8.7 (6.4, 10.9)	9.1 (7.5, 10.7)	11.0 (8.8, 13.2)	--
Model 1 (hazard ratio, 95% CI)	1.00	1.18 (0.87, 1.60)	1.47 (1.15, 1.89)	0.003
Model 2 (hazard ratio, 95% CI)	1.00	1.17 (0.86, 1.58)	1.37 (1.09, 1.73)	0.014
Model 3 (hazard ratio, 95% CI)	1.00	1.14 (0.84, 1.55)	1.24 (0.96, 1.59)	0.134
Model 4 (hazard ratio, 95% CI)	1.00	1.18 (0.87, 1.59)	1.13 (0.88, 1.44)	0.712
Model 5 (hazard ratio, 95% CI)	1.00	1.16 (0.86, 1.55)	1.08 (0.85, 1.38)	0.962
Model 6 (hazard ratio, 95% CI)	1.00	1.16 (0.86, 1.56)	1.14 (0.90, 1.44)	0.589
Model 4: Exclude prevalent diabetes, cardiovascular disease, cancer	1.00	1.15 (0.69, 1.92)	0.96 (0.63, 1.47)	0.406
Model 5: Exclude deaths in year 1	1.00	1.20 (0.89, 1.61)	1.11 (0.86, 1.42)	0.917
**Diseases of the circulatory system**				
Unweighted no. deaths/no. at risk	46/2088	74/3367	70/1895	--
Unweighted person-years	11,892	19,218	10,393	--
Age-adjusted rate/1,000 PY (95% CI)	3.3 (2.0, 4.6)	2.7 (1.8, 3.5)	3.2 (2.4, 4.1)	--
Model 1 (hazard ratio, 95% CI)	1.00	0.82 (0.48, 1.40)	1.05 (0.72, 1.53)	0.388
Model 2 (hazard ratio, 95% CI)	1.00	0.84 (0.48, 1.46)	1.00 (0.68, 1.48)	0.606
Model 3 (hazard ratio, 95% CI)	1.00	0.86 (0.49, 1.54)	0.96 (0.62, 1.48)	0.880
Model 4 (hazard ratio, 95% CI)	1.00	0.90 (0.50, 1.64)	0.87 (0.55, 1.36)	0.578
Model 5 (hazard ratio, 95% CI)	1.00	0.96 (0.54, 1.71)	0.88 (0.53, 1.45)	0.555
Model 6 (hazard ratio, 95% CI)	1.00	0.98 (0.56, 1.71)	0.96 (0.60, 1.52)	0.845
Model 4: Exclude prevalent diabetes, cardiovascular disease, cancer	1.00	1.07 (0.46, 2.47)	1.65 (0.75, 3.62)	0.186
Model 5: Exclude deaths in year 1	1.00	1.00 (0.55, 1.84)	0.84 (0.48, 1.46)	0.418

*CI *= confidence interval; *PY *= person-years

Model 1 is adjusted for age

Model 2 is adjusted for age, gender, race or ethnicity, educational attainment

Model 3 is adjusted for variables in model 2 plus smoking status, leisure-time physical activity, Healthy Eating Index score, and alcohol consumption

Model 4 is adjusted for variables in model 3 plus health status and health insurance coverage

Model 5 is adjusted for variables in model 4 plus histories of diabetes, cardiovascular disease, and cancer

Model 6 is adjusted for variables in model 5 plus body mass index, systolic blood pressure, and concentrations of high-density lipoprotein cholesterol, non-high-density lipoprotein cholesterol, and HbA1c

When participants with chronic conditions (diabetes, cardiovascular disease, and cancer) were excluded, the hazard ratios were only slightly lower (Table 5). The maximally-adjusted model for deaths from diseases of the circulatory system suggested a trend of increasing risk with increasing screen time. Because of the limited number of deaths, however, the estimates should be cautiously interpreted.

**Table 5:** Sample sizes, rates, and hazard ratios for mortality from all-causes and diseases of the circulatory system among participants aged > = 20 years after excluding prevalent self-reported diabetes, cardiovascular disease, and cancer, National Health and Nutrition Examination Survey 1999-2002-2006

	Screen time (hours per day)						
	
	< 1	1	2	3	4	> = 5	P for trend
**All-causes**							
Unweighted no. deaths/no. at risk	21/744	32/967	48/1565	46/1010	22/611	41/719	--
Unweighted person-years	4232	5617	9096	5843	3534	3963	--
Age-adjusted rate/1,000 PY (95% CI)	5.4 (1.1, 9.6)	5.9 (3.0, 8.9)	5.0 (3.8, 6.1)	8.1 (5.3, 10.8)	4.1 (2.1, 6.2)	8.6 (5.8, 11.5)	--
Model 1 (hazard ratio, 95% CI)	1.00	1.04 (0.46, 2.34)	1.02 (0.48, 2.14)	1.62 (0.63, 4.12)	0.80 (0.33, 1.94)	1.85 (0.86, 3.98)	0.051
Model 2 (hazard ratio, 95% CI)	1.00	1.06 (0.46, 2.40)	1.00 (0.48, 2.09)	1.60 (0.65, 3.95)	0.77 (0.33, 1.81)	1.71 (0.82, 3.57)	0.091
Model 3 (hazard ratio, 95% CI)	1.00	1.08 (0.48, 2.45)	0.98 (0.48, 2.01)	1.49 (0.61, 3.64)	0.72 (0.30, 1.72)	1.45 (0.69, 3.06)	0.325
Model 4 (hazard ratio, 95% CI)	1.00	1.12 (0.50, 2.53)	1.03 (0.51, 2.09)	1.48 (0.62, 3.52)	0.71 (0.30, 1.68)	1.33 (0.65, 2.74)	0.592
Model 5 (hazard ratio, 95% CI)	1.00	1.11 (0.49, 2.53)	1.01 (0.49, 2.08)	1.47 (0.62, 3.50)	0.70 (0.30, 1.64)	1.29 (0.63, 2.64)	0.634
**Diseases of the circulatory system**							
Unweighted no. deaths/no. at risk	6/744	8/967	10/1565	15/1010	9/611	15/719	--
Unweighted person-years	4232	5617	9096	5843	3534	3963	--
Age-adjusted rate/1,000 PY (95% CI)	1.1 (0.1, 2.2)	1.1 (0.0, 2.2)	0.6 (0.0, 1.4)	2.3 (0.7, 3.9)	1.3 (0.4, 2.3)	3.5 (1.0, 6.1)	--
Model 1 (hazard ratio, 95% CI)	1.00	1.04 (0.19, 5.62)	0.52 (0.11, 2.41)	1.46 (0.42, 5.05)	0.89 (0.25, 3.17)	2.68 (0.83, 8.70)	0.071
Model 2 (hazard ratio, 95% CI)	1.00	1.05 (0.19, 5.74)	0.57 (0.12, 2.73)	1.58 (0.45, 5.50)	0.96 (0.27, 3.43)	2.58 (0.80, 8.32)	0.074
Model 3 (hazard ratio, 95% CI)	1.00	1.10 (0.21, 5.78)	0.58 (0.12, 2.76)	1.55 (0.42, 5.68)	0.93 (0.26, 3.28)	2.45 (0.72, 8.35)	0.101
Model 4 (hazard ratio, 95% CI)	1.00	1.22 (0.22, 6.96)	0.67 (0.14, 3.15)	1.77 (0.50, 6.33)	1.07 (0.29, 3.93)	2.58 (0.75, 8.82)	0.081
Model 5 (hazard ratio, 95% CI)	1.00	1.47 (0.24, 8.81)	0.75 (0.16, 3.42)	2.04 (0.54, 7.70)	1.43 (0.34, 6.07)	3.17 (0.86, 11.63)	0.042

*CI *= confidence interval; *PY *= person-years

Model 1 is adjusted for age

Model 2 is adjusted for age, gender, race or ethnicity, educational attainment

Model 3 is adjusted for variables in model 2 plus smoking status, leisure-time physical activity, Healthy Eating Index score, and alcohol consumption

Model 4 is adjusted for variables in model 3 plus health status and health insurance coverage

Model 5 is adjusted for variables in model 5 plus body mass index, systolic blood pressure, and concentrations of high-density lipoprotein cholesterol, non-high-density lipoprotein cholesterol, and HbA1c

## Discussion

Like other studies, the present study found that U.S. adults spent considerable time watching television or videos or using a computer. However, the present study offers little support for the hypothesis that prolonged screen time may increase mortality from all-causes and diseases of the circulatory system. Furthermore, control for confounding may be a particularly challenging issue in studies of screen time and mortality.

Previous prospective studies have reported variable findings about the relationship between the amount of television watching and mortality [13-16]. In an analysis of data from the Aerobics Center Longitudinal Study, 7744 men were followed for 21 years during which time 377 men died from cardiovascular disease [14]. The hours per week of screen time were divided into quartiles. After adjusting for age, smoking, alcohol use, family history of cardiovascular disease, body mass index, physical activity, and self-reported hypertension, diabetes, and hypercholesterolemia, screen time was not significantly related to mortality from cardiovascular disease (quartile 2: HR = 1.02, 95% CI = 0.74, 1.42; quartile 3: HR = 1.27, 95% CI = 0.90, 1.78; quartile 4: HR = 0.96, 95% CI = 0.68, 1.36; p for trend = 0.94).

In the Australian Diabetes, Obesity, and Lifestyle Study, 8800 adults aged ≥ 25 years were followed on average for 6.6 years during which time 284 participants died (87 from cardiovascular disease) [13]. Compared to adults who watched television < 2 h/day, those watching 2- < 4 h/day, and 4 or more hours per day had a significantly increased risk for mortality from all-causes (HR = 1.13, 95% CI = 0.87, 1.36; HR = 1.46, 95% CI = 1.04, 2.05, respectively) and cardiovascular disease (HR = 1.19, 95% CI = 0.72, 1.99; HR = 1.80, 95% CI = 1.00, 3.25, respectively). The hazard ratios were adjusted for age, gender, education, smoking status, energy intake, alcohol use, diet quality index, waist circumference, hypertension, total cholesterol, high-density lipoprotein cholesterol, triglycerides, lipid-lowering medications, and glucose tolerance status.

In the European Prospective Investigation into Cancer and Nutrition Norfolk Study, 13,197 men and women with a mean age of 61.5 years were followed for a mean of 9.5 years and 1270 participants died (373 from cardiovascular disease) [16]. After adjusting for age, gender, education, smoking status, alcohol use, medications for hypertension and dyslipidemia, history of diabetes, family history of CVD and cancer, and physical activity, the hazard ratio per hour/day of watching television was 1.05 (95% CI: 1.01, 1,09) for all-cause mortality and 1.08 (95% CI: 1.01, 1.16) per hour/day for mortality from cardiovascular disease.

Recently, a fourth prospective study using data from the Scottish Health Survey was published [15]. During a mean follow-up of 4.3 years, 325 deaths (215 CV deaths) occurred among 4,512 men and women aged ≥ 35 years. Compared to participants who watched television, used a computer, or played video games < 2 h per day, increased risks for all-cause mortality and fatal and nonfatal diseases of the circulatory system were noted for those who did so for 2**- **< 4 h per day (all-cause mortality aHR = 1.14, 95% CI = 0.80**-**1.62; diseases of the circulatory system aHR = 2.23 95% CI = 1.31**-**3.80) and ≥ 4 h per day (all-cause mortality aHR = 1.48, 95% CI = 1.04**-**2.13; diseases of the circulatory system aHR = 2.25 95% CI = 1.30**-**3.89). The risk estimates were adjusted for age, gender, ethnicity, body mass index, smoking, social class, long-standing illness, marital status, diagnosed diabetes and hypertension, occupational physical activity, and physical activity.

Of the four studies, including the present study, that examined the associations between screen time and all-cause mortality, only the present study failed to produce a significant association. Nevertheless, the adjusted hazard ratio of 1.31 in the present study for participants reporting ≥ 5 h per day of screen time is not inconsistent with the published estimates of screen time discussed above. The reasons for the dissonant findings are not entirely clear. All four studies used self-reported information to assess screen time although the questions in the studies differed. The follow-up times of the other three studies ranged from 4.3 years to 9.5 years compared to 5.8 years in the present study. Death status in all studies was determined through linkages to vital statistics.

Of the five studies, including the present one, that explored the associations between screen time and cardiovascular outcomes, two studies including the present one failed to report significant associations [14]. A meta-analysis of previous prospective studies of screen time and all-cause mortality and fatal or nonfatal cardiovascular disease estimated that the summary relative risk per 2 h of screen time per day was 1.13 (95% CI = 1.07, 1.18) for all-cause mortality and 1.15 (95% CI = 1.06, 1.23) for fatal or nonfatal cardiovascular disease [23]. Adding the results from the present study to the data shown in the meta-analysis changes the fixed-effects summary estimate of relative risk per 2 h of screen time to 1.12 (95% CI: 1.07, 1.17; test for heterogeneity p = 0.741) and 1.14 (95% CI: 1.06, 1.22; test for heterogeneity p = 0.736), respectively. Thus, the totality of the current evidence continues to suggest that prolonged screen time poses a threat to health.

Several additional prospective studies examined the relationships between sedentary behavior, which included watching television in some studies, and mortality [24-26]. Although these three studies did not focus on screen time per se, they did find that excessive sedentary behavior, primarily in the form of sitting, showed a small increase in the risk of mortality from all-causes and cardiovascular disease.

Strengths of the study included the population-based sample that is representative of adults in the United States (excellent external validity), decent sample size, good response rates, and inclusion of a broad spectrum of potential confounding variables and of several potential cardiometabolic mediators that were objectively measured. However, these strengths deserve to be balanced against various limitations of the study. Like many other studies, the amount of screen time was self-reported. The questions used in the survey were not specifically validated for this study. However, self-reported screen time has been shown to correlate with other measures in expected ways and, in the present study, correlated as expected with a number of anthropometric and clinical variables. A review of the reliability and validity of self-reported television viewing and other sedentary behavior described acceptable reliability but variable validity [27].

Because the question or questions used to measure screen time constitute a critical aspect of the prospective studies, it is interesting to note that all prospective studies to date differ in their assessment of screen time. The Aerobics Center Longitudinal Study assessed average weekly time spent watching television [14]; the Australian Diabetes, Obesity, and Lifestyle Study assessed total time spent watching television during the previous 7 days excluding time that the television was turned on but was not being watched [13]; the European Prospective Investigation into Cancer and Nutrition Norfolk Study asked about time spent watching television on weekdays and weekends [16]; and the Scottish Health Survey assessed the time spent watching television, using a computer, or playing video games on weekdays and weekends [15].

Consequently, some of the heterogeneity in the findings of the four studies examining the relationship between screen time and all-cause mortality and five studies of screen time and cardiovascular mortality might be attributable to the differences in questionnaires used to assess screen time. First, three of the previous studies assessed only the time spent watching television or watching videos [13,14,16], whereas the third study, like the present one, assessed the time spent watching television, using a computer, or playing video games [15]. However, one of the first three studies failed to produce a significant association between screen time and cardiovascular disease whereas of the two studies that included time spent watching television, using a computer, or playing video games, one study reported a significant association [15] and the present study did not. Thus, it seems unlikely that this aspect of the questions about screen time explains the variation among studies. Nevertheless, the health effects of prolonged television viewing, which has been related to other unhealthy lifestyle behaviors notably unhealthy dietary elements, conceivably differ from those of prolonged use of a computer or playing video games.

Second, one study that reported significant associations between screen time and mortality from all-causes and cardiovascular disease attempted to limit screen time to the time that participants specifically watched television and not to the time that the television was turned on [13]. However, among the remaining four studies that did not involve this methodological twist, two studies produced positive findings for mortality from cardiovascular disease [15,16], and two studies, including the present one, produced negative findings [14]. This aspect of the study questionnaires does not appear to readily explain the different findings of the studies.

Third, some studies measured screen time separately on weekdays and weekend days [15,16] although not all studies were clear about this aspect of the exposure assessment. In the present study, screen time was not separately assessed for days during the week and weekend.

In the present study, participants reported sizeable amounts of screen time. The Nielsen Company data show that Americans spend on average about 35 h per week watching television, 2 h of time shifted television, and 4 h on the internet [1]. The self-reported data from the present study suggests that the most commonly reported and median reported screen time was around 2 h, an estimate that is lower than the 2.7 h reported in the 2010 American Time Use Survey. Thus, underreporting of the amount of screen time by the NHANES participants is a possibility. If participants tended to underreport the true amount of screen time, the hazard ratios in the present study may have been underreported if participants at increased risk for mortality were shifted into the referent group thus raising the baseline risk of the referent group.

With 5.8 years of follow-up time, the duration of the present study is at the shorter end of the distribution, which ranges from 4.3 to 21 years. The study of 4.3 years found a significant association, whereas the study of 21 years did not. Consequently, it is unclear whether the duration of a study affects the chances of observing a significant result. As in virtually all observational studies, the results may have changed had additional known or unknown confounders been included in the analyses.

## Conclusion

The amount of screen time did not significantly predict mortality from all-causes and diseases of the circulatory system in the present study. Nevertheless, the totality of the evidence from prospective studies continues to suggest that prolonged screen time adversely affects health. Because only a limited number of prospective studies have addressed the issue of whether screen time predicts mortality, additional such studies are needed to clarify and quantify any relationship.

Disclaimer: The findings and conclusions in this article are those of the author and do not necessarily represent the official position of the Centers for Disease Control and Prevention.

## Competing interests

The author declares that they have no competing interests.

## Authors' contributions

EF performed all work pertaining to this manuscript.

## Pre-publication history

The pre-publication history for this paper can be accessed here:

http://www.biomedcentral.com/1471-2458/12/70/prepub
